# Mastoid emissary vein, mastoid emissary foramen, and mastoid emissary canal: anatomy, variability, imaging, and clinical implications

**DOI:** 10.1007/s00276-026-03912-z

**Published:** 2026-06-01

**Authors:** Mugurel Constantin Rusu, Răzvan Costin Tudose, Alexandra Diana Vrapciu

**Affiliations:** 1 Division of Anatomy, Department 1, Faculty of Dentistry, “Carol Davila” University of Medicine and Pharmacy, 050474 Bucharest, Romania; 2 Research Department, “Dr. Carol Davila” Central Military Emergency Hospital, 010825 Bucharest, Romania

**Keywords:** Mastoid emissary vein, Mastoid emissary foramen, Mastoid emissary canal, Cone-beam CT, Multidetector CT, Pulsatile tinnitus, Osia, Retrosigmoid approach

## Abstract

**Purpose:**

The mastoid emissary vein (MEV), mastoid emissary canal (MEC), and mastoid emissary foramen (MEF) are common but variably reported structures of the mastoid-posterior fossa region. Existing literature is fragmented across osteological, imaging-based, and clinical studies, with inconsistent prevalence and morphometric estimates, and with limited integration of anatomical variation into surgical and radiological decision-making. This SANRA-guided narrative review aimed to synthesise the anatomy, variability, imaging assessment, and clinical implications of MEV/MEC/MEF.

**Methods:**

This SANRA-guided narrative review synthesised anatomical, imaging, and clinical studies identified through PubMed/MEDLINE, Scopus, and Google Scholar from database inception through March 2026. Eligible studies included dry-skull, cadaveric, CBCT, MDCT/HRCT, and case-based reports addressing morphology, morphometrics, prevalence, or clinical relevance. Owing to methodological heterogeneity, findings were synthesised narratively.

**Results:**

Across osteological and imaging studies, MEF/MEC prevalence was generally high but heterogeneous, with frequent unilateral or bilateral multiplicity. Most canals/foramina were small, whereas a minority were markedly enlarged and potentially relevant to surgical bleeding, collateral venous drainage, or venous pulsatile tinnitus. Enlarged MEVs have been implicated in selected cases of venous pulsatile tinnitus, with reported treatments including conservative management, surgical clipping/ligation, and endovascular or percutaneous occlusion. In mastoid and posterior fossa surgery, prominent MEVs have been reported as potential sources of difficult venous bleeding. In paediatric Osia implantation, larger preoperative MEV calibre correlated with intraoperative bleeding.

**Conclusions:**

MEV/MEC/MEF should be assessed systematically on preoperative imaging, including presence, number, calibre, and relationships to surgical landmarks. Consistent reporting may reduce avoidable haemorrhagic and diagnostic complications.

## Introduction

Emissary veins are valveless venous conduits that traverse cranial foramina/canals to link the intracranial dural venous sinuses with extracranial venous networks. The mastoid emissary vein (MEV) is among the most clinically encountered emissary veins in the mastoid/posterior auricular region. It is relevant to posterior cranial fossa and mastoid surgery because its calibre and number are variable, and its course lies close to the sigmoid sinus. Injury can result in brisk haemorrhage and may be challenging to control in a narrow operative corridor [22, 23]. Conversely, enlargement of the MEV, mastoid emissary canal (MEC), or mastoid emissary foramen (MEF; also termed mastoid foramen in parts of the anatomical literature) can be symptomatic, including as a venous cause of pulsatile tinnitus (PT) [1, 24, 27, 53].

In an anatomical synthesis from Bergman’s Comprehensive Encyclopedia of Human Anatomic Variation, emissary veins are described as valveless connections between extracranial veins and intracranial venous sinuses that may act as pressure-equalising safety valves, particularly during elevated intracranial pressure or altered venous outflow. They are more commonly encountered in children, with emissary foramina typically larger during childhood, and they can be identified on MR, CT, and angiographic studies when clinically relevant [49].

In the era of modern radiology, haemostatic materials, and multidisciplinary lateral skull-base approaches, MEV variability is not usually a major limitation to surgery on its own. Its practical relevance is selective: it becomes important when an enlarged, multiple, dehiscent, or functionally collateral emissary channel intersects a planned mastoid, retromastoid, or lateral skull-base operative corridor.

Despite this clinical importance, the literature remains fragmented. Reported MEF/MEC prevalence varies substantially across dry-skull, cadaveric, CBCT, MDCT/HRCT, and routine CT studies. Morphometric values are difficult to compare because studies differ in whether they measure the external foramen, internal opening, canal diameter, venous calibre, or per-side versus per-patient prevalence. In addition, many clinically relevant observations are derived from case reports or small surgical series, so anatomical variability, imaging appearance, and operative implications are not consistently integrated. These limitations make it difficult to translate isolated morphometric data into practical reporting or surgical-planning recommendations.

Therefore, this SANRA-guided narrative review aims to synthesize the anatomy, variability, imaging assessment, and clinical implications of the MEV, MEC, and MEF, with particular attention to how these structures should be interpreted in preoperative imaging, otologic and posterior fossa surgery, and evaluation of venous pulsatile tinnitus.

## Methods

This narrative review was conducted following the SANRA (Scale for the Assessment of Narrative Review Articles) guidelines for narrative reviews [6]. No protocol registration was performed as narrative reviews are not eligible for PROSPERO registration.

A literature search was performed in PubMed/MEDLINE, Scopus, and Google Scholar from database inception to 5th March 2026. No automatic restrictions were applied for publication date, study type, or human-only records, because the review aimed to include historical anatomical series, dry-skull studies, cadaveric dissections, imaging studies, and clinical case reports. The initial search was not restricted by language; however, articles were included in the final synthesis only when the full text was available in English or a reliable English translation was accessible. The PubMed/MEDLINE search strategy used the following Boolean combination of terms: (“mastoid emissary vein” OR “mastoid emissary veins” OR “mastoid emissary canal” OR “mastoid emissary canals” OR “mastoid emissary foramen” OR “mastoid emissary foramina” OR “mastoid foramen” OR “mastoid foramina”). The same conceptual search strategy was adapted for Scopus and Google Scholar, in accordance with the syntax requirements of each database. In addition, the reference lists of eligible articles and relevant reviews were manually screened to identify further studies not retrieved by the electronic database search.

**Fig. 1:**
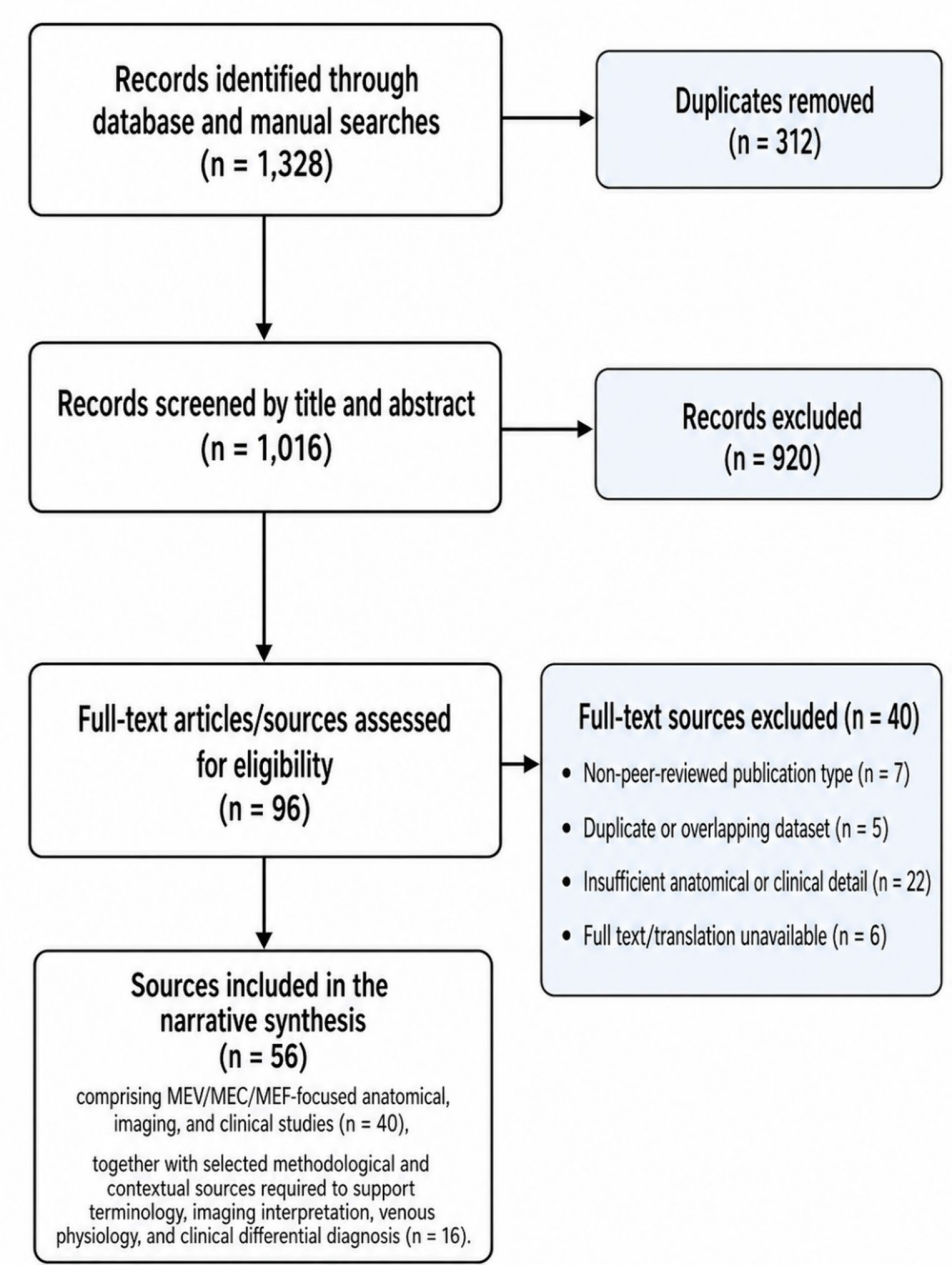
Flow diagram of literature identification, screening, eligibility assessment, and inclusion

The search identified 1,328 records/sources; after removal of 312 duplicates, 1,016 records were screened, 96 full-text articles/sources were assessed for eligibility, 40 were excluded, and 56 were included in the final narrative synthesis, comprising MEV/MEC/MEF-focused anatomical, imaging, and clinical studies (40), together with selected methodological and contextual sources required to support terminology, imaging interpretation, venous physiology, and clinical differential diagnosis (16). The selection process is summarized in Fig. 1.

Inclusion criteria: (1) original research articles (anatomical, imaging-based, or clinical studies) reporting on the morphology, morphometrics, prevalence, or clinical relevance of the mastoid emissary vein, mastoid emissary canal, or mastoid emissary foramen; (2) case reports and case series documenting clinical presentations or interventions related to MEV/MEC/MEF; (3) studies utilizing dry skulls, cadaveric specimens, or imaging modalities (CBCT, MDCT, HRCT, MRI, CT angiography/venography); (4) studies published in peer-reviewed journals.

Exclusion criteria were: (i) abstracts, conference proceedings, or editorials without original data; (ii) studies focusing exclusively on other emissary veins without mastoid emissary vein data; (iii) non-English publications without available translation; (iv) duplicate publications of the same dataset.

For study selection and data extraction, two authors (MCR, RCT) independently screened titles and abstracts for relevance. Full-text articles meeting eligibility criteria were retrieved and assessed. Disagreements were resolved by consensus among the authors (ADV). Data extraction was performed using a standardized form that captured: study design; sample characteristics (sample size, population, modality); prevalence data; morphometric measurements (foramen/canal diameter, length, distances to anatomical landmarks); multiplicity patterns; intracranial communication patterns; and clinical outcomes, where reported.

Given the heterogeneity of study designs, populations, imaging modalities, and measurement protocols across included studies, a quantitative meta-analysis was not feasible. Data were synthesized narratively and presented in summary tables stratified by study design (anatomical/dry skull vs. imaging-based) and imaging modality (CBCT vs. MDCT/HRCT). Comparative tables were constructed to facilitate cross-study comparison of prevalence rates, morphometric parameters, and multiplicity classifications.

Because this was a narrative review including heterogeneous anatomical, imaging, clinical, case-based, and contextual sources, no formal methodological quality score was assigned. Instead, eligible studies were descriptively appraised for methodological features relevant to interpretation. Anatomical and imaging studies provide the strongest basis for prevalence and morphometric discussion, although they remain limited by variable definitions, modality-specific detection bias, and inconsistent measurement sites. By contrast, several clinical implications, including severe intraoperative haemorrhage, treatment of MEV-related pulsatile tinnitus, collateral-flow preservation, and implant-related bleeding, are supported mainly by case reports or small retrospective series. Accordingly, the clinical statements in this review should be read as risk-awareness and reporting recommendations rather than formal evidence-based management guidelines.

### Anatomy and terminology

The MEV typically courses through the MEC within the mastoid part of the temporal bone, communicating intracranially with the sigmoid sinus and draining extracranially into posterior auricular and/or occipital venous tributaries and the suboccipital venous plexus; downstream drainage can extend into vertebral venous pathways [5, 14, 28, 33, 36]. A MEF is the extracranial bony aperture of this pathway; multiple foramina can be present on one side, and in dry skulls, the MEF is most often located near the occipitomastoid suture or on the posterior margin of the mastoid process [9, 16]. Besides the emissary vein, the MEF may transmit a meningeal branch of the occipital artery [16, 18, 20].

Bergman’s encyclopedia chapter on emissary veins summarises the MEV as traversing the MEC to connect the transverse or sigmoid sinus to posterior auricular and/or occipital venous pathways that may continue to the vertebral venous plexus [16, 49].

In this review, the term MEV is used for a transosseous emissary vein that traverses the mastoid portion of the temporal bone through the MEC and exits through the MEF. Under this definition, an intracranial connection with the sigmoid sinus, transverse-sigmoid junction, or, less commonly, the adjacent transverse sinus region may represent a variant proximal connection of the same mastoid emissary pathway if the extracranial exit is through the MEF/MEC. Conversely, venous channels that do not traverse the MEC/MEF should not be labelled as MEV, even when they participate in posterior fossa collateral drainage; they are better interpreted as petrosquamosal, occipital, condylar, or other skull-base emissary pathways according to their course.

Classic anatomical work emphasised that the mastoid foramen is most often located in the mastoid portion of the temporal bone but may also lie within the masto-occipital (occipitomastoid) suture or, more rarely, within the occipital bone close to that suture. In Knott’s series of 44 skulls (88 sides), the foramen was suture-located in 14% of cases, and the corresponding vessels passed through an occipital-bone foramen in 3/88 sides; the foramen/vein were larger on the right in 34/44 skulls, larger on the left in 6/44, and nearly equal in 4/44 [25].

### Developmental and physiological considerations

The MEV has been described as a residual embryonic venous tract connecting the sigmoid sinus to extracranial venous pathways [12, 27]. The MEV is valveless and can support bidirectional flow; this is clinically relevant when the vessel serves as a collateral outflow route in the setting of altered central venous drainage, intracranial hypertension, or high-flow states, or when elevated venous pressure increases calibre [14, 32, 42]. From a practical standpoint, the greatest impact is on procedural risk when enlarged and on symptom generation when haemodynamics are audible (e.g., PT) [1, 27, 53].

Embryological descriptions of the craniocervical venous system suggest that the transverse sinus continues into the ventral cardinal vein (future internal jugular vein), but that secondary bypass pathways exist between the lateral sinus and the external jugular system, specifically the mastoid emissary vein (via the mastoid foramen) and the petrosquamous sinus. These emissary channels tend to atrophy, leaving a small remnant of the MEV that anastomoses with the suboccipital plexus, which is described as an origin of the vertebral veins [29].

Physiological studies demonstrate marked postural dependence of cerebral venous drainage. It was reported that the predominance of the jugular veins in cerebrovenous drainage is largely limited to the supine position. In contrast, in the erect position, the vertebral venous system represents the major outflow pathway [51]. San Millán Ruíz et al. similarly noted that moving from the supine to the upright position favours venous outflow into vertebral venous systems rather than internal jugular veins and show that mastoid and occipital emissary veins, together with the anterior, lateral, and posterior condylar veins, provide key connections between posterior fossa dural sinuses, the internal jugular system, and cervical vertebral venous plexuses [41].

These transosseous emissary connections can be conceptualised as part of the suboccipital venous network. In their microsurgical anatomy study, Arnautović et al. describe the suboccipital venous plexus as continuing inferiorly into the deep cervical vein and communicating with the transverse–sigmoid sinus via the mastoid emissary and occipital veins, positioning the MEV as one component of a larger suboccipital cavernous sinus complex [4].

Skull-base venous crossroads may mobilise multiple emissary channels depending on flow direction and resistance. Literature on hypoglossal canal dural arteriovenous fistulas similarly places the anterior condylar confluence and other emissary veins, including the MEV, within a network linking posterior fossa dural sinuses to internal and external vertebral venous plexuses [43].

### Morphology and prevalence

Across osteological, cadaveric, and imaging studies, MEF/MEC presence is generally common, but reported prevalence varies widely according to population, modality, definition, and unit of analysis (Table 1). Dry-skull and cadaveric series usually report high prevalence, whereas routine head CT may under-detect smaller canals compared with direct anatomical inspection. CBCT, MDCT, and HRCT studies generally provide better depiction of the osseous MEF/MEC, while contrast-enhanced venous imaging is required when the venous lumen, flow direction, or collateral role is clinically relevant. A recent systematic review and meta-analysis by Alzir et al. pooled 21 studies comprising 8,689 skull sides and estimated the overall prevalence of the mastoid emissary foramen at 74%, with a pooled mean diameter of 2 mm [3]. These data support the view that the MEF/MEC is common but variably expressed, and that individual prevalence estimates should be interpreted in relation to the examined structure, imaging modality, and reporting criteria.

**Table 1:** Prevalence and multiplicity of MEF in included anatomical and imaging studies

Study	Design/Modality	Sample	Prevalence/presence	Multiplicity highlights
Knott [25]	Anatomical skull series	44 skulls (88 sides)	Not absent on either side in the examined series; absence noted in additional dissections (5 sides)	Masto-occipital suture location 14%; occipital bone 3/88; right larger 34/44
Boyd [7]	Dry skulls	1478 skulls	68%	-
Falk [19]	Dry skulls	51 skulls	90%	-
Wysocki [52]	Dry skulls	Not reported	94%	-
Reis [36]	Cadaveric	18 sides	89%	-
Louis [28]	Cadaveric	200 sides	98% (R), 72% (L)	Multiplicity types I–V reported
Kim [23]	Dry skulls	106 skulls	83.7%	15% > 2.5 mm; 4.3% > 4 mm
Murlimanju [32]	Dry temporal bones	96 temporal bones	91.7%	Single 62.5%; double 22.9%; triple 6.2%
Demirpolat [14]	MDCT	248 patients (496 sides)	MEC present 92.3%	Absent–triple MEF; bilateral MEC 74.6%
Ozen [33]	HRCT (COM vs controls)	62 COM mastoids; 100 control mastoids	MEC present 87.1% (COM) vs 80% (controls)	Accessory MEC 61.8% (COM) vs 31.3% (controls)
Yurdabakan [54]	CBCT	472 patients	82%	Bilateral MEC 55%; unilateral MEF 27%; none 18%
Temiz [48]	CBCT	240 patients	88.1%	Bilateral 66.1%
Zhou [56]	Cadaveric + routine CT + intraop	15 cadavers (30 sides); 51 patients (102 sides CT; 51 sides intraop)	90% (cadaver); 49% (CT); 94% (intraop)	External openings: 1 (56.7%), 2 (30%), 3 (3.3%); single internal opening
Duque-Parra [16]	Dry skull halves	206 halves (103 skulls)	94.17%	Up to 8 foramina/half
Chaiyamoon [9]	Dry skulls + cadavers	44 sides (dry skulls) + 10 sides (cadavers)	90.9%	Type 0 absent 9.09%; type I 50%; type II 22.73%; type III 13.64%; type IV 4.55%
Erturk [18]	MDCT	357 patients	92.15%	-
Rozumenko [37]	Retrosigmoid cohort; pre-/post-operative CT	20 patients (10 mapped; 10 controls)	MEV canal used for mapping; 1–2 external openings in the mapped group	Two external openings in 50% of mapped cases
Donofrio [15]	HRCT + cadaveric correlation + RSA cohort	100 HRCT; 54 RSA; 4 temporal bones	MEF type 0 (absent): 17–18% per side; MEC absent: 7% (patients)	MEC bilateral 64%; unilateral 29% (patients)
Russo [38]	Contrast-enhanced CT ± MRI (paediatric AOM cohort)	282 AOM (58 ACOM; 224 AUOM) + 282 controls	Unusually enlarged MEV: ACOM 2/58; AUOM 3/224; controls 5/282; jugular bulb protrusion: 17/58; 16/224; 37/282	Variants defined for risk analysis; multiplicity not reported

*CBCT* cone-beam computed tomography, *HRCT* high-resolution computed tomography, *MDCT* multidetector computed tomography, *COM* chronic otitis media

A qualitative point from the Bergman synthesis is that multiple small mastoid (or related posterior fossa) emissary veins can converge toward a single external foramen, and that an enlarged mastoid emissary foramen does not necessarily imply transmission of a proportionately enlarged vein. The mastoid emissary foramen has also been reported to be less frequent in females than in males in some anatomical studies [49].

### Morphometrics and relationships to landmarks

Reported MEF and MEC dimensions vary substantially because studies measure different targets, including the external foramen, internal opening, bony canal, canal midpoint, or venous lumen (Table 2). Most reported diameters are in the low-millimetre range. Larger canals or foramina, particularly in the approximate 3–4 mm range or above, should be interpreted as potential risk markers rather than as automatic predictors of bleeding or symptoms. Their significance depends on surgical corridor, relationship to mastoid air cells and the sigmoid sinus, and whether the MEV contributes to collateral venous drainage. Discrepancies between “foramen,” “canal,” and “vein” measurements are also modality-driven: dry-skull studies and CBCT/CT primarily assess the osseous channel, whereas contrast-enhanced CT, CT venography, MR venography, and Doppler ultrasound are needed to characterize the venous component.

**Table 2:** Selected morphometric parameters from included studies

Study	Parameter	Estimate	Notes
Kim [23]	Mean MEF diameter	1.64 mm (maximum 7 mm)	-
	Proportion of large MEF	15% > 2.5 mm; 4.3% > 4 mm (hemiskulls)	-
Demirpolat [14]	Mean MEF diameter	Right 1.92 ± 1.02 mm (0.1–6.3); left 1.84 ± 0.98 mm (0.1–4.7)	-
	Mean MEC diameter	Right 1.58 ± 0.86 mm (0.1–5.2); left 1.48 ± 0.79 mm (0.1–5.1)	-
	Proportion of large MEF	> 4 mm: 2.4% (R), 2.2% (L); > 3 mm: 9.5% (R), 8.5% (L)	-
Temiz [48]	Mean MEF diameter (CBCT)	2.4 ± 0.9 mm	-
	Mean MEC diameter (CBCT)	2.1 ± 0.8 mm	-
Yurdabakan [54]	Mean MEF diameter (CBCT)	3.39 ± 1.48 mm	-
	Mean MEC diameter (CBCT)	2.05 ± 1.06 mm	-
	MEF-to-asterion distance	Right 22.46 ± 5.06 mm; left 23.03 ± 4.66 mm	-
	MEF-to-mastoid-tip distance	Right 29.60 ± 5.61 mm; left 28.94 ± 6.45 mm	-
Erturk [18]	MEF diameter range (MDCT)	Right 0.6–5.0 mm (mean 1.80); left 0.6–4.4 mm (mean 1.96)	-
Duque-Parra [16]	MEF diameter (dry skull)	Median 1.82 mm (0.28–7.3) across 383 foramina	-
Hampl [22]	External opening diameter	1.3 ± 0.7 mm (0.2–6.0)	-
	Internal opening diameter	1.7 ± 1.3 mm (0.2–9.0)	-
Louis [28]	MEF-to-asterion distance	19.9 mm (17.2–23.3)	-
	MEF-to-mastoid-tip distance	35.4 mm (32.8–37.4)	-
Reis [36]	Distance from MEV to the sinodural angle	18.94 mm	-
	MEV length	11.77 mm (6.5–17.8)	-
Chaiyamoon [9]	Non-connecting MEF external opening diameter	1.17 ± 0.57 mm (0.36–2.56)	-
	Connecting MEF length	13.59 ± 4.19 mm (7.14–25.17)	-
	Connecting MEF internal opening diameter	3.05 ± 0.97 mm (0.84–5.43)	-
	Internal opening to the sigmoid sinus junction	15.72 ± 3.63 mm (9.58–21.71)	-
	MEV course (sagittal plane)	Ascending 9.52%; almost horizontal 57.14%; descending 33.33%	-
Louis [28]	MEV length (origin to termination)	7.2 cm (3.8–11.8)	-
	Cadaver external opening diameter	1.84 ± 0.85 mm (0.78–4.57)	-
Zhou [56]	Cadaver: external opening to asterion	22.33 ± 4.94 mm (13.56–31.74)	-
	Cadaver: external opening to mastoid tip	28.23 ± 4.02 mm (18.82–37.24)	-
	Cadaver straight intra-mastoid length	11.93 ± 3.58 mm (6.45–19.32)	-
	Cadaver: internal opening to the TS–SS junction	17.35 ± 2.90 mm (12.58–22.57)	-
	Cadaver proportion large calibre	> 2.5 mm: 16.7%; > 4 mm: 6.7%	-
	CT external opening diameter	2.06 ± 1.03 mm (0.70–5.61)	Routine head CT bone window
	CT proportion large calibre	> 2.5 mm: 17.6%; > 4 mm: 3.9%	-
	MEV canal: external–internal opening distance	11.90 ± 1.18 mm (9.40–14.10)	CT-derived; mapping cohort
Rozumenko [37]	External opening to the projected posterior SS border	9.36 ± 2.17 mm (6.3–13.20)	Used to map the posterior SS border
	External opening count	1–2; two openings 50% (5/10)	Mapped subgroup
	MEF–MAC distance	R: 9.8 ± 6.5 mm; L: 10.3 ± 5.4 mm	MAC = most posterior mastoid air cell (Han-based grading)
Donofrio [15]	mMEC–MAC distance	R: 3.5 ± 3.9 mm; L: 3.2 ± 3.5 mm	mMEC = midpoint of MEVC
	MEF/MEC posterior to MACs	MEF 99.4%; MEC 88.0%	When within MACs, air-cell entry risk increases

*SS* sigmoid sinus

### Intracranial communication patterns

The typical intracranial connection is with the sigmoid sinus sulcus. In a dry-skull series probing 383 foramina, 55.87% communicated with the sulcus for sigmoid sinus, while 38.64% showed no intracranial communication by the authors’ testing method; less common communications opened toward the lateral part of the posterior cranial fossa, the transverse–sigmoid junction, and rare communications to the sulcus for the greater petrosal nerve or to diploic emissary pathways within the temporal bone [16]. It was found that, regardless of the number of external MEF openings, these converged to a single internal opening in the sulcus of the sigmoid sinus; it was also documented a variant in which two external MEFs shared one internal opening with bifurcation of the MEV [9]. Other authors similarly described internal openings relative to the sigmoid sinus sulcus and provided distances to otologic landmarks relevant to posterior fossa approaches [22]. In a large cadaveric series, mastoid and occipital emissary veins most often formed a confluent venous system (85%), with single-vessel patterns in 15% [28].

Imaging case literature also illustrates that a markedly dilated MEV can participate in complex collateral drainage and show atypical intracranial connections. In a patient with tinnitus, HRCT and CT venography depicted an enlarged MEC (~ 7.5 mm) forming an intramastoid loop with two posterior fossa openings, while MR venography characterised the associated venous component, bilateral petrosquamosal sinuses, posterior condylar and occipital emissary pathways, and transverse-sigmoid sinus hypoplasia/atresia [11]. A further CTA-based case documented a large extracranial fenestration of the MEV: a 6.6 mm vein exiting through a single mastoid foramen, divided 2.2 mm distal to the foramen and rejoined 2.33 cm inferiorly, with three communicating veins to the suboccipital venous plexus and distal continuation as the deep cervical vein [39]. Such patterns reinforce the need to evaluate not only intracranial communications and intramastoid course, but also the extracranial segment and global venous outflow context before MEV sacrifice or occlusion.

**Fig. 2:**
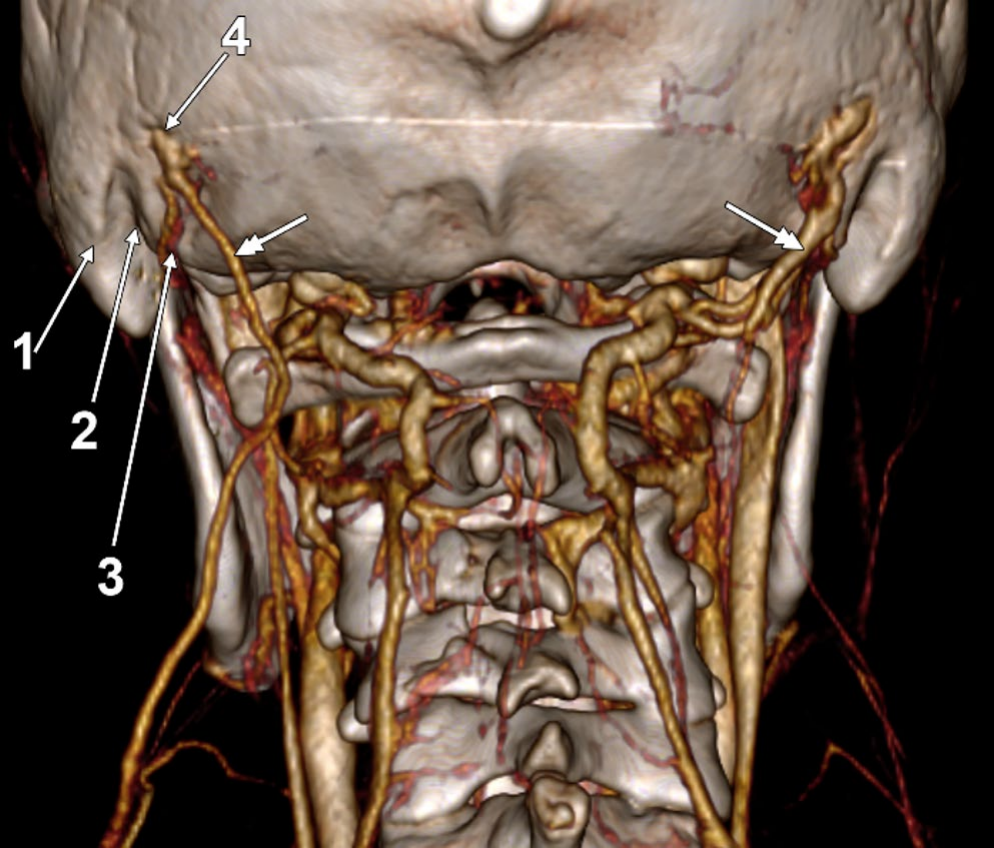
Bilateral mastoid emissary veins (double-headed arrows). CT angiogram, three-dimensional volume rendering. Posterior-inferior view. 1. mastoid process; 2. mastoid/digastric notch; 3. occipital artery sulcus; 4. mastoid foramen

Historical work on venous sinus variation also supports the concept that emissary pathways can substitute for diminished dural sinuses. Knott described two instances of near absence of the right lateral sinus, with only a small venous canal (~ 1.5 mm diameter) following the expected sinus course as far as the mastoid foramen, through which it then exited [25]. In the same skull series, the posterior condylar emissary vein was present bilaterally in 13/44 skulls (as evidenced in Fig. 2). It was more frequently present on the right than the left (21 vs 10 cases), underscoring that MEV variants are often best interpreted within a broader posterior fossa emissary network [25].

Beneath the jugular foramen, the anterior condylar confluence constitutes a venous crossroad connecting dural sinuses with both the internal jugular and vertebral venous systems; case-based literature on hypoglossal canal dural arteriovenous fistulae emphasises the complexity of these craniocervical junction connections and the postural dependence of venous drainage, reinforcing that prominent emissary channels (including the MEV) should be interpreted within a broader condylar–vertebral outflow context [10, 41].

### Imaging considerations

CBCT and MDCT/HRCT can depict the bony canal/foramen and, when contrast-enhanced CT or MR venography is available, can help characterise the venous component. CBCT studies emphasise preoperative reporting of the presence, number, diameter, and location of MEC/MEF relative to the occipitomastoid suture and asterion [48]. In otologic populations, HRCT can also identify accessory MECs and demonstrate calibre enlargement associated with chronic inflammatory ear disease [33]. For retrosigmoid planning, Chaiyamoon et al. (2024) emphasised correlating the MEF/MEV course on CT with the intended burr-hole position because MEF/MEV can be used as superficial landmarks for localising the deeper-lying sigmoid sinus [9]. Across older imaging literature, reported MEV visualisation/prevalence rates vary by modality and definitions (e.g., 63% on MRI, 82% on axial CT, 77.7% on CT angiography, and 70.5% on MRI) [21, 26, 35, 41]. Zhou et al. (2023) showed that routine bone-window head CT underdiagnoses MEF/MEV compared with cadaveric dissection but still identifies a clinically relevant subset of large-calibre canals and depicts intraosseous course patterns (straight vs. curved), supporting its use for operative risk stratification in retrosigmoid planning. Conversely, in otologic surgery, even preoperative contrast-enhanced MRI may fail to reveal a surgically significant large MEV, underscoring that non-visualisation does not exclude the risk of haemorrhage [8]. The broader emissary-vein literature also cautions that foramen calibre alone should not be assumed to reflect venous calibre [49].

At the craniocervical junction, contrast-enhanced three-dimensional fast spoiled gradient–recalled acquisition in the steady state (3D fast SPGR) MR imaging with fat suppression has been used to delineate the suboccipital cavernous sinus and its connections with anterior, lateral and posterior condylar veins and the anterior internal vertebral venous plexus, providing an imaging framework for understanding collateral routes between posterior fossa dural sinuses, emissary veins, and cervical venous plexuses [47]. Endovascular case literature further illustrates that these pathways may preferentially drain into deep cervical veins (e.g., suboccipital cavernous sinus drainage to the deep cervical vein), which can be exploited for alternative transvenous access in selected lesions [45].

Temporal bone CT evaluation benefits from thin-slice acquisition and bone-window review, with axial and coronal reconstructions usually sufficient for identifying the MEF/MEC and its relationship to mastoid air cells, sutures, and the sigmoid sinus region [5]. Oblique reformats, including Stenvers-type planes, should not be presented as superior or routinely necessary for MEV assessment. Their potential value is limited to selected cases in which the canal has an oblique course, its relationship to mastoid air cells is unclear, or differentiation from a suture or fracture line is difficult. Intravenous contrast is not required to identify the bony canal, but contrast-enhanced CT, CT venography, MR venography, or Doppler ultrasound may be useful when the venous lumen, flow, collateral drainage, or pulsatile tinnitus mechanism must be evaluated.

### Do not misjudge the mastoid emissary canal/foramen as exclusively “a vein”

Although the MEC/MEF is commonly discussed in venous terms (i.e., as the bony conduit for the MEV), it should not be interpreted as a purely venous structure. Syed and colleagues emphasised that the MEC transmits both the MEV and a branch of the occipital artery, meaning that the canal and foramen represent a neurovascular corridor rather than an isolated venous channel [46]. This distinction matters clinically and radiologically. First, an enlarged foramen and canal may be encountered incidentally on CBCT/CT and must be recognised as a normal variant to avoid mislabelling it as a destructive “cortical defect” or other pathology; careful review of borders, contents, and surrounding structures (with multiplanar and 3D reconstructions where available) is recommended to differentiate anatomical variation from disease [46]. Second, failure to recognise a prominent foramen and canal (and the contents) may lead to iatrogenic, potentially life-threatening bleeding during procedures in the mastoid region [46]. Accordingly, when reporting mastoid emissary structures, the radiologist should explicitly note that the canal/foramen may transmit both arterial and venous components, and should document unusually enlarged channels as relevant procedural-risk anatomy [46].

### Surgical relevance and complications

The surgical relevance of the MEV is determined less by its presence alone than by its calibre, number of openings, relationship to the operative corridor, and role in venous drainage. Small MEVs are usually incidental. Enlarged canals or foramina, multiple external openings, dehiscence into mastoid air cells, or evidence of collateral venous drainage should be considered risk modifiers during mastoidectomy, retrosigmoid craniotomy, bone-conduction implant placement, and selected otologic or endovascular procedures [2, 8, 9, 13, 15, 22, 23, 37, 44, 56].

Although much of the available MEV surgical literature concerns retrosigmoid craniotomy, the same anatomical principles apply to wider lateral skull-base procedures involving the posterior mastoid, retrolabyrinthine region, sigmoid sinus, jugular bulb, or petro-occipital corridor. Thus, MEF/MEC assessment should be approach-specific: in retrosigmoid approaches, the main issue is the relationship to the burr hole, sigmoid sinus border, and mastoid air cells; in translabyrinthine, transotic, transcochlear, presigmoid/combined petrosal, combined retrosigmoid-transotic, and petro-occipital trans-sigmoid approaches, the main relevance is during mastoidectomy, retrolabyrinthine or presigmoid drilling, flap elevation, and exposure around the sigmoid-jugular region [30, 31, 55].

Several mechanisms explain the operative risk. The MEV may communicate with the sigmoid sinus or transverse-sigmoid region and may show bidirectional flow, so injury to a sizeable channel can cause rapid venous bleeding in a narrow field [22, 23, 36, 56]. Multiple external foramina may converge toward a single internal opening, meaning that the external anatomy may underestimate the intracranial connection [9, 56]. A large MEV may also represent an important collateral pathway when jugular venous outflow is restricted, or the contralateral transverse-sigmoid system is hypoplastic or stenotic; in such cases, preservation or venous-outflow assessment should be considered before elective sacrifice [11, 28, 42, 44].

The evidence supporting specific haemorrhage thresholds remains limited. A diameter of around 3.5–4 mm has been used in surgical reports as a practical alert range, and Kim et al. reported that 15% of hemiskulls had foramina > 2.5 mm and 4.3% had foramina > 4 mm [23]. Surgical planning should integrate canal calibre, number of external openings, intraosseous course, mastoid pneumatization, relationship to the planned drilling margin, and the broader venous outflow pattern [9, 15, 22, 37, 56]. Haemostatic measures such as bone wax may be effective, but should be used cautiously because sigmoid sinus thrombosis from bone-wax migration and rare ischaemic complications after emissary-vein sacrifice have been described [28, 36, 42, 56].

**Fig. 3:**
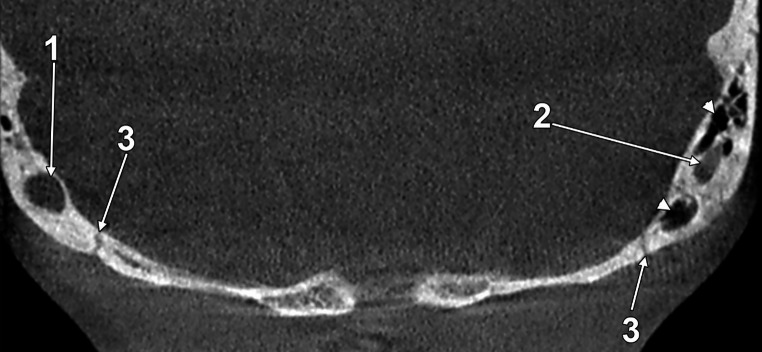
Coronal CBCT slice, anterior view. Original evidence. On the right side, a 6.08 mm-calibre mastoid canal is found (1). On the left side, a mastoid canal (2) of 3.89 mm courses among mastoid air cells (arrowheads). The occipitomastoid sutures are indicated bilaterally (3)

The MEC coursing through the mastoid process must also be distinguished from adjacent mastoid air cells on preoperative imaging (Fig. 3).

In mastoidectomy and otologic surgery, the main risk is unexpected bleeding from an overlooked enlarged MEV or dehiscent canal [2, 8, 23]. In paediatric Osia implantation, a larger preoperative MEV calibre was associated with intraoperative bleeding in a small series, supporting review of MEV calibre and course when preoperative imaging is available [13]. This principle may be relevant to other posterior mastoid bone-conduction systems when the implant bed overlaps the MEF/MEC region, although direct device-specific evidence beyond Osia remains limited. In paediatric acute otomastoiditis, enlarged emissary channels are uncommon but may coexist with sigmoid-jugular variants and inflammatory venous complications; they should therefore be interpreted as part of the broader temporal bone and dural venous assessment rather than as an isolated causal factor [38].

### Pulsatile tinnitus associated with enlarged MEV

Case reports and small series indicate that MEV-related pulsatile tinnitus usually involves an enlarged or dehiscent MEV/MEC, often with symptom reduction during ipsilateral jugular compression or focal mastoid compression. Reported treatments include conservative management, surgical clipping or ligation, endovascular coiling, and direct percutaneous embolisation. However, treatment decisions require correlation with a venous clinical phenotype and confirmation that the MEV is not a critical collateral outflow pathway (Table 3). In routine temporal bone CT workup of pulsatile tinnitus, other vascular variants at the skull base, including high or dehiscent jugular bulb and jugular bulb diverticulum, should also be assessed to avoid misattribution of symptoms [5, 50].

**Table 3:** MEV-related pulsatile tinnitus and intervention outcomes in the included case literature

Study	Patient	Key imaging finding	Intervention	Outcome
Lee [27]	44F	Inner foramen ~ 4.5 mm (left)	Conservative	Adapted; no intervention
Abdalkader [1]	61F	MEV up to 5 mm (right)	Endovascular coiling	Complete resolution; improved at 6 months
Kim [24]	45F	MEV canal; mid ~ 2.5 mm; sinus connection ~ 3 mm	Surgical clipping/ligation	Improved; no recurrence > 2 years
Yang [53]	61 M	Dilated MEC: 4.0 mm (R), 2.6 mm (L)	Conservative	No surgery (tolerable symptoms)
Sahoo [40]	36 M	Dilated MEV ~ 4 mm; prominent MEC; drains to vertebral venous plexus	Direct percutaneous puncture + detachable coil embolisation	Immediate tinnitus resolution
Chauhan [11]	30F	Enlarged MEC (~ 7.5 mm) with intramastoid loop; 2 posterior fossa openings; bilateral petrosquamosal sinuses	Conservative (masker + nortriptyline)	Symptomatically improved

Osseovenous dehiscence involving the MEV has been defined on high-resolution temporal bone CT as direct contact between the MEV and a mastoid air cell without intervening bone [17]. In the small series by Eliezer et al., this CT finding alone did not predict treatment response; complete symptom resolution after selective MEV embolisation occurred only in patients with a clinical venous pulsatile tinnitus phenotype, defined by interruption or decrease of tinnitus during ipsilateral jugular compression [17]. This distinction is important because an enlarged or dehiscent MEV may be anatomically conspicuous but clinically incidental. When clinically relevant, the absence of bony separation may facilitate transmission of venous flow noise through mastoid air cells, whereas the surgical relevance relates mainly to the risk of laceration during mastoid drilling [17, 34].

Before clipping, ligation, coiling, or direct puncture embolisation, the wider venous outflow context should be assessed because enlarged emissary veins may contribute materially to cranial drainage when jugular or transverse-sigmoid outflow is limited [40, 49]. Sahoo et al. reported successful direct percutaneous puncture and detachable coil embolisation of a dilated MEV after other treatments had failed, but they also emphasised careful patient selection and confirmation of adequate transverse-sigmoid sinus filling before occluding an emissary drainage route [40]. Thus, MEV-related pulsatile tinnitus should be diagnosed only when imaging findings, clinical compression manoeuvres, and exclusion of alternative vascular causes converge.

### Practical reporting checklist for radiology / preoperative planning

**Fig. 4:**
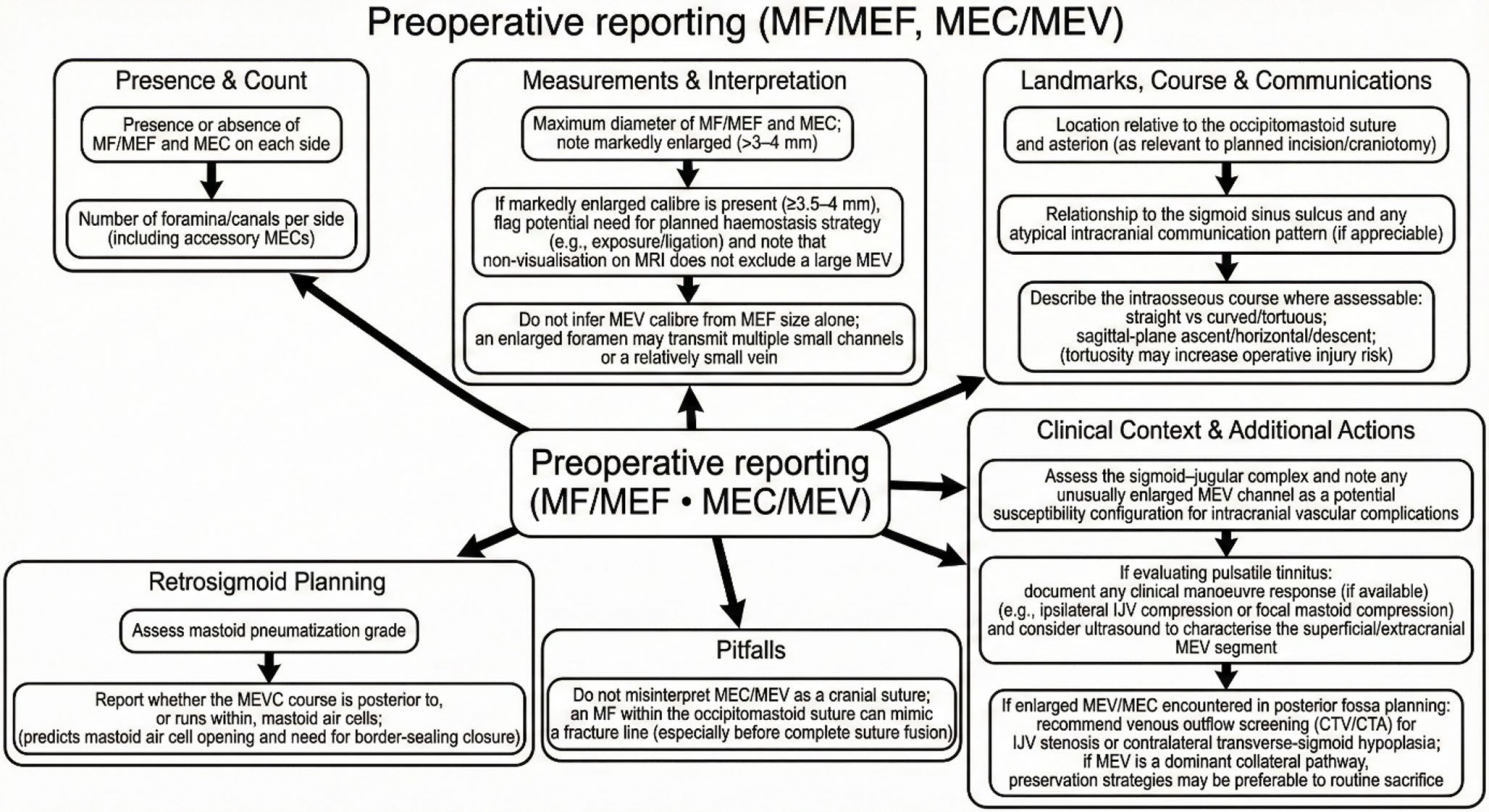
Practical reporting imaging checklist related to the presence of the mastoid emissary foramen (MEF), mastoid emissary canal (MEC), and mastoid emissary vein (MEV)

Drawing on patterns observed across the included studies, preoperative radiological assessment of the mastoid region should be systematically structured to address several key elements (Fig. 4). The report should first document the presence or absence of the MEF and MEC on each side, along with the number of foramina or canals per side, including any accessory structures, since the number may range from absent to quadruple [14, 28]. The maximum diameter of the MEF/MEC should be recorded, but size should be interpreted as one component of risk rather than as an isolated determinant. Particular attention should be paid to markedly enlarged channels, especially in the approximate 3–4 mm range or above. A 3.5-mm value has been used in limited surgical literature as a practical alert threshold, but it should not be presented as a validated universal definition of clinical significance. The location relative to the occipitomastoid suture and asterion should be specified when relevant to the planned incision or craniotomy, given that the MEF is most commonly situated at the occipitomastoid suture in approximately 52% of cases [9, 54]. Care must be taken not to misinterpret the MEC or MEV as a cranial suture, as an MEF within the occipitomastoid suture can mimic a fracture line, particularly before complete suture fusion.

The relationship to the sigmoid sinus sulcus and any atypical intracranial communication patterns should be described where appreciable, since the MEV serves as a reliable landmark for estimating sigmoid sinus position during transcondylar and retrosigmoid approaches [9, 14, 42]. In retrosigmoid surgical planning, the grade of mastoid pneumatisation should be assessed. The report should indicate whether the mastoid emissary venous channel courses posterior to the mastoid air cells or runs within the mastoid air cells, as this predicts the likelihood of mastoid air cell opening and the consequent need for border-sealing closure [9]. The intraosseous course should be described where assessable, classifying it as straight or curved/tortuous, and noting sagittal-plane orientation (ascending, horizontal, or descending), since curved MEVs with tortuous courses are more likely to be damaged during retrosigmoid craniotomy [9, 42].

When the MEF/MEC appears markedly enlarged, particularly in the approximate 3.5–4 mm range or above, the report may alert the surgeon to consider a planned haemostasis strategy, while emphasizing that calibre alone does not determine risk and that non-visualisation on MRI does not exclude a surgically relevant MEV [14, 23]. Importantly, MEV calibre should not be inferred from MEF size alone, as an enlarged foramen may transmit multiple small channels or a relatively small vein, and rudimentary foramina not pierced by vessels have been described [9, 14, 36]. In patients being evaluated for pulsatile tinnitus, any clinical manoeuvre response should be documented, if available, such as ipsilateral internal jugular vein compression or focal mastoid compression. Ultrasound may be considered to characterise the superficial extracranial MEV segment, as Doppler sonography has proven valuable in confirming MEV-related tinnitus [1, 27]. When an enlarged MEV or MEC is encountered during posterior fossa surgical planning, venous outflow screening with CTV or CTA for internal jugular vein stenosis or contralateral transverse-sigmoid hypoplasia should be recommended; if the MEV constitutes a dominant collateral pathway, preservation strategies may be preferable to routine sacrifice, since ligation of the MEV may result in venous ischaemia and haemorrhage when it serves as a major outflow route [14, 28, 42].

### Limitations and future directions

This review is limited by its narrative design and by the heterogeneity of the available evidence. Many prevalence and morphometric studies use different definitions, measurement sites, imaging modalities, and units of analysis, which limits direct pooling. The surgical literature specifically addressing MEV/MEC/MEF is disproportionately centred on retrosigmoid craniotomy and mastoidectomy, while evidence for extended lateral skull-base approaches is more inferential and should be interpreted cautiously. Several clinical implications, including haemorrhage thresholds, implant-related bleeding, preservation of collateral venous drainage, and treatment of MEV-related pulsatile tinnitus, are supported mainly by case reports or small series. Therefore, the practical recommendations in this review should be understood as imaging and surgical risk-awareness suggestions rather than formal management guidelines.

Future anatomical work should prioritise clinically oriented multimodal correlation rather than additional isolated prevalence estimates. Useful studies should combine thin-slice bone-window CT or CBCT assessment of MEF/MEC with contrast-enhanced venous imaging or Doppler ultrasound of the MEV, use standardized measurement points, report observer reliability, distinguish osseous canal size from venous lumen size, and correlate enlarged or dehiscent channels with surgical bleeding, pulsatile tinnitus phenotype, implant placement, mastoid pneumatisation, and collateral venous drainage.

## Data Availability

The dataset used and analyzed during the current study is available from the corresponding author upon reasonable request.

## Abbreviations

3D: Three-dimensional
3D fast SPGR: Three-dimensional fast spoiled gradient-recalled acquisition in the steady state
ACOM: Acute complicated otomastoiditis
AOM: Acute otomastoiditis
AUOM: Acute uncomplicated otomastoiditis
CBCT: Cone-beam computed tomography
COM: Chronic otitis media
CT: Computed tomography
CTA: Computed tomography angiography
CTV: Computed tomography venography
F: Female
HRCT: High-resolution computed tomography
L: Left
M: Male
MAC: Mastoid air cell
MDCT: Multidetector computed tomography
MEC: Mastoid emissary canal
MEF: Mastoid emissary foramen
MEV: Mastoid emissary vein
mMEC: Midpoint of the mastoid emissary venous channel
MR: Magnetic resonance
MRI: Magnetic resonance imaging
PT: Pulsatile tinnitus
R: Right
RSA: Retrosigmoid approach
SS: Sigmoid sinus
TS: Transverse sinus
